# Trauma and posttraumatic stress disorder in South Africa: analysis from the South African Stress and Health Study

**DOI:** 10.1186/1471-244X-13-182

**Published:** 2013-07-03

**Authors:** Lukoye Atwoli, Dan J Stein, David R Williams, Katie A Mclaughlin, Maria Petukhova, Ronald C Kessler, Karestan C Koenen

**Affiliations:** 1 Department of Mental Health, School of Medicine, Moi University College of Health Sciences, Eldoret, Kenya; 2 Department of Psychiatry and Mental Health, University of Cape Town, Cape Town, South Africa; 3 Department of Society, Human Development and Health, Harvard School of Public Health, Boston, MA, USA; 4 Department of Psychiatry, Division of General Pediatrics, Children’s Hospital Boston, Harvard Medical School, Boston, MA, USA; 5 Department of Health Care Policy, Harvard Medical School, Boston, MA, USA; 6 Department of Epidemiology, Mailman School of Public Health, Columbia University, New York, NY, USA

**Keywords:** Posttraumatic stress disorder, Trauma, South Africa

## Abstract

**Background:**

South Africa’s unique history, characterised by *apartheid*, a form of constitutional racial segregation and exploitation, and a long period of political violence and state-sponsored oppression ending only in 1994, suggests a high level of trauma exposure in the general population. The aim of this study was to document the epidemiology of trauma and posttraumatic stress disorder (PTSD) in the South African general population.

**Methods:**

The South African Stress and Health Study is a nationally representative survey of South African adults using the WHO’s Composite International Diagnostic Interview (CIDI) to assess exposure to trauma and presence of DSM-IV mental disorders.

**Results:**

The most common traumatic events were the unexpected death of a loved one and witnessing trauma occurring to others. Lifetime and 12-month prevalence rates of PTSD were 2.3% and 0.7% respectively, while the conditional prevalence of PTSD after trauma exposure was 3.5%. PTSD conditional risk after trauma exposure and probability of chronicity after PTSD onset were both highest for witnessing trauma. Socio-demographic factors such as sex, age and education were largely unrelated to PTSD risk.

**Conclusions:**

The occurrence of trauma and PTSD in South Africa is not distributed according to the socio-demographic factors or trauma types observed in other countries. The dominant role of witnessing in contributing to PTSD may reflect the public settings of trauma exposure in South Africa and highlight the importance of political and social context in shaping the epidemiology of PTSD.

## Background

South Africa is a developing country with a history characterised by past constitutional racial segregation and exploitation in the form of *apartheid* that gave way to a non-racial democracy only in 1994 [1]. This transition was achieved by a protracted liberation struggle, characterized by political violence and state-sponsored oppression. After apartheid, high levels of often criminal interpersonal violence continued, fuelled by rapid urbanization and ongoing socioeconomic disparities, that resulted in a high level of trauma exposure, over 80% in some studies [1-6].

The present study reports on the prevalence of trauma exposure and risk of PTSD associated with potentially traumatic events (PTEs) in the South African Stress and Health Survey (SASH), the first nationally representative study of mental disorders in Africa [7]. Although two prior reports on trauma and PTSD using the SASH data have been published [8,9], this is the first report using the random event method to describe the full range of PTE exposure and PTSD in the South African general population.

The previous reports either focussed only on nonspecific psychological distress [8] rather than posttraumatic stress disorder, or on interpersonal violence [9] rather than the entire range of PTEs. Both reports, like many others before them, also used the ‘worst event’ method of identifying PTEs, rather than the ‘random event’ method we used in the present study.

In most community surveys, respondents usually report lifetime exposure to a very large number of PTEs, making it impossible to carry out a separate assessment of PTSD for each PTE experienced by every respondent. This problem is typically addressed in community epidemiologic surveys by asking respondents to nominate the worst PTE they ever had in their lifetime and using that one PTE as the focus of PTSD assessment. This approach often overestimates conditional risk of PTSD because worst traumas are atypical and presumably have a higher risk of PTSD compared with more typical traumas [10-12]. In the present study, this problem was resolved by assessing PTSD for two PTEs: the one nominated by the respondent as their worst lifetime PTE and another computer-generated random PTE selected from among the respondent’s other lifetime PTEs. Both the worst lifetime PTE and the randomly selected PTE were then assessed for PTSD symptoms, generating a dataset that accurately reflected the occurrence of PTEs in the sample population.

Others, including Norris et al have also used the random event method before, although they did not utilise computerised selection of the random event [11]. Computerised selection of the random event to be assessed for PTSD is meant to minimise the risk of bias and increase the probability that the selected PTEs reflect the actual prevalence in the population.

Our approach therefore allows us to provide a more accurate account than in previous studies of the relative importance of difference traumas in accounting for PTSD in the South African population.

## Methods

### Sample

The South African Stress and Health (SASH) study [7] was carried out between January 2002 and June 2004 as part of the WHO World Mental Health Surveys [13] to determine the prevalence and identify risk factors for mental disorders in South Africa. The rationale and survey methods have been described in previous publications [7,8] and are only summarized briefly here.

The SASH protocol was reviewed and approved by the Human Subjects Research Ethics Committees of the University of Michigan, Harvard Medical School and the Medical University of South Africa (MEDUNSA) [7]. Informed consent was obtained from all participants before conducting interviews.

The study population comprised adult South Africans residing both in households and hostels. Individuals living in institutions such as hospitals, prisons, mental health institutions and military bases were excluded from the study.

Respondents were selected using a multi-stage area probability sample design. In the first stage, a stratified probability sample of primary sampling areas was selected. These areas are roughly equivalent to counties in the US or the UK, and the size was based on the 2001 South African Census of Enumeration Areas (EAs). The EAs were sampled with probabilities proportionate to their population size. In the second stage, a random sample of 5 households was selected within each EA. The third stage consisted of a random selection of a single adult respondent in each selected household.

A maximum of three attempts were made to contact each selected respondent, and the study achieved an overall response rate of 85%. The demographic characteristics of the sample have been described previously [8]. Briefly, the total sample of 4,315 adults had a higher proportion of women (58.6%) and Blacks (79.7%), although other racial groups were represented (10.4% Colored, 7.2% White, and 2.7% Indian/Asian). The classification of *Colored* in South Africa represents a heterogeneous racial group of mixed ancestry as discussed elsewhere [14]. Use of these groups in the current paper is done not with the intention of reifying them, but rather with an awareness of the ongoing disparities that remain across these groups even post-apartheid, their possible differential impact on the experience of trauma and PTSD [9]. Further, one half was married, most were unemployed (69.2%), had less than 12 years of education (62.7%), and lived in urban areas (59.7%).

### Measures

#### Trauma exposure

The SASH assessed lifetime occurrence of the 27 PTEs included in the WHO composite International Diagnostic Interview (CIDI) DSM-IV PTSD module [15]. PTEs were categorized into 8 classes as follows: war events (combat, relief worker in a war zone, civilian in a war zone, civilian in a region of terror, refugee and purposely injured, tortured or killed someone), physical violence (physical abuse by caregiver, physical assault by spouse or romantic partner, physical assault by someone else, mugged or threatened with a weapon, and kidnapped), sexual violence (raped, sexually assaulted and stalked), accidents (toxic chemical exposure, automobile accident, other life-threatening accident, natural disaster, man-made disaster, and a life-threatening illness), unexpected death of a loved one, network events involving others (having a child with a serious illness, traumatic event occurring to a loved one, and accidentally causing serious injury or death) and witnessing (witnessing a death, seeing a dead body or someone seriously hurt, seeing atrocities, and witnessing domestic violence). The final category of other included an additional question inquired about other PTEs not included in the CIDI list and a final open-ended question obtained information about qualifying PTEs that respondents did not report because of embarrassment (coded as ‘Private events’).

For each of the trauma items, the possible responses were ‘Yes’, ‘No’, ‘Don’t Know’ and ‘Refuse to Answer. Additionally, for each PTE occurrence, the respondents indicated how old they were when the event occurred, and how long it lasted.

#### PTSD assessment

DSM-IV requires PTSD to be assessed in relation to exposure to a qualifying PTE. However, just like in other community surveys of trauma and PTSD, most SASH respondents reported lifetime exposure to multiple PTEs and some respondents reported exposure to a very large number of PTEs. We therefore used the random event method as described in the introduction, additionally weighting the data on symptoms associated with the latter PTEs by the number of lifetime PTEs each respondent reported having. We were thus able to construct a weighted PTE-level dataset that accurately represents all PTEs that ever occurred to all respondents. Unlike datasets based only on information about worst PTEs, this weighted dataset of randomly selected PTEs can be used to obtain unbiased estimates of PTSD conditional prevalence and distribution across all PTEs in the population.

DSM IV PTSD Criterion A2 (Response) was considered met if the respondent endorsed any of three questions about whether, at the time of PTE exposure, he/she felt terrified or very frightened, helpless, shocked, or horrified. This was followed with structured questions about re-experiencing (criterion B), avoidance-numbing (criterion C), arousal (criterion D), duration (criterion E), and clinically significant distress or impairment (criterion F).

For each DSM IV PTSD symptom cluster (B, C and D) the respondent was asked how soon after the event the symptoms started, how many days, weeks, months or years the symptoms continued and how often the symptoms occurred per month when they were at their most frequent or intense.

#### Socio-demographic correlates

Six socio-demographic variables were included in the analysis: gender, age, marital status, education, employment status and race. Age consisted of four categories (in years): 18–29, 30–44, 45–59, and 60 or older. Marital status was categorised into three groups: married, previously married and never married. Education was classified depending on number of years of formal schooling into four categories: Low (0-1 year), low-average (2-7 years), high-average (8-12 years) and high (13 or more years). Employment status consisted of four categories: employed, homemaker, retired and other (including unemployed and students). Race consisted of the four standard categories in South Africa: Black, White, Indian/Asian, and Colored. Socio-demographic variables with multiple categories were dummy coded for analytic purposes (reference groups include age 60+, Married, High education, Employed and White race).

### Analysis methods

Prevalence of PTE exposure and conditional prevalence of PTSD given PTE exposure were examined using cross-tabulations. A series of four logistic regression models [16] were then used to examine the predictors of lifetime and 12-month PTSD.

For lifetime PTSD, the first model examined its socio-demographic predictors in the population. The second model examined the socio-demographic predictors of exposure to any traumatic event in the total sample while the third model examined predictors of lifetime PTSD among those with exposure to at least one event. The final model was similar to the third, but additionally controlled for the type of PTEs and prior exposure to PTEs.

For 12-month PTSD, the first model also examined the socio-demographic predictors in the total population, while the second one examined predictors of 12-month PTSD among those with PTE exposure. The third model was similar to the second but additionally controlled for type of PTEs and prior exposure to PTEs. The final model examined the predictors of 12-month PTSD among those with lifetime PTSD, controlling for both PTE exposure and prior PTEs.

The logistic regression coefficients and their standard errors were exponentiated and are reported here as odds-ratios (ORs) with 95% confidence intervals. To adjust for the weighting and clustering of the SASH data, standard errors (SE) were estimated using the Taylor series method [17] implemented in the SUDAAN software system [18]. Multivariate significance was evaluated with Wald χ2 tests based on design-corrected coefficient variance-covariance matrices. Statistical significance was consistently evaluated using .05 level two-sided tests.

## Results

### PTE exposure

Prevalence (standard error) of exposure to at least one lifetime PTE was 73.8% (1.15) in the total sample, while the average person exposed to any lifetime PTE reported an average of 4.3 occurrences, for a total of approximately 13,700 lifetime PTEs experienced by the 4,315 SASH respondents (Table 1). The PTE class reported by the highest proportion of respondents was unexpected death of a loved one (39.2%) followed by physical violence (37.6%), accidents (31.9%), and witnessing (29.5%).

**Table 1:** Prevalence of trauma exposure in the South Africa Stress and Health Study (N = 4315)

**Event type**	**Prevalence %**	**SE**	**Mean no. of occurrences**^**a**^	**SE**	**Proportion of traumas in population**^**b**^	**SE**
**War events**	**12.2**	**0.87**	**1.5**	**0.06**	**5.8**	**0.54**
Combat	2.6	0.35	1	0	0.8	0.1
Relief worker	2.1	0.33	1	0	0.7	0.1
Civilian in war zone	3.6	0.67	1	0	1.1	0.21
Civilian in region of terror	7.1	0.73	1	0	2.2	0.23
Refugee	1.6	0.28	1	0	0.5	0.09
Purposely injured, tortured, or killed someone	0.9	0.17	1.5	0.16	0.5	0.09
**Physical violence**	**37.6**	**0.98**	**1.8**	**0.04**	**20.9**	**0.60**
Beaten up by caregiver	12	0.82	1	0	3.8	0.26
Beaten up by partner	7.9	0.48	1	0	2.5	0.14
Beaten up by someone else	12.4	0.61	1.5	0.06	5.9	0.34
Mugged or threatened with a weapon	18.3	0.79	1.4	0.04	8.4	0.36
Kidnapped	1.2	0.21	1	0	0.4	0.07
**Sexual violence**	**7.6**	**0.56**	**1.8**	**0.08**	**4.4**	**0.34**
Raped	2.1	0.27	1.3	0.07	0.9	0.13
Sexually assaulted	1.6	0.24	1.8	0.17	0.9	0.17
Stalked	4.5	0.43	1.9	0.11	2.7	0.25
**Accidents**	**31.9**	**1.06**	**1.9**	**0.05**	**19.6**	**0.57**
Toxic chemical exposure	3.2	0.40	2.3	0.19	2.3	0.38
Automobile accident	13.2	0.64	1.2	0.03	5.1	0.24
Other life threatening accident	5.6	0.42	1.4	0.07	2.5	0.25
Natural disaster	4.1	0.51	1.3	0.06	1.7	0.21
Man-made disaster	2.8	0.27	1.3	0.12	1.2	0.15
Life-threatening illness	13.2	0.76	1.6	0.05	6.8	0.36
**Unexpected death of loved one**	**39.2**	**1.22**	**1.6**	**0.04**	**19.6**	**0.57**
**Network events**	**14.1**	**0.74**	**1.6**	**0.07**	**6.9**	**0.47**
Child with serious illness	8.1	0.51	1.4	0.05	3.5	0.27
Traumatic event to loved one	5.8	0.41	1.4	0.09	2.5	0.25
Accidentally caused serious injury or death	1.9	0.28	1.6	0.26	1	0.22
**Witnessing**	**29.5**	**1.29**	**2.2**	**0.09**	**20.5**	**0.96**
Witnessed death/dead body, saw someone hurt	28.5	1.29	2.0	0.07	18.1	0.89
Saw atrocities	3.9	0.51	1.9	0.15	2.4	0.37
**Others**	**6.4**	**0.52**	**1.1**	**0.02**	**2.2**	**0.18**
Some other event	3.0	0.37	1	0	0.9	0.12
Private event	3.9	0.40	1	0	1.2	0.13
**Total with any event**	**73.8**	**1.15**	**4.3**	**0.11**	**100**	**0**

^a^Mean number of occurrences among respondents with any PTE vary significantly across the eight PTE classes (χ_5_ = 489.8, p < .001 as well as across the 28 individual PTE types (χ_18_ = 1698, p < .001). ^b^Events in this class as percentage of all traumatic events.

**Figure 1:**
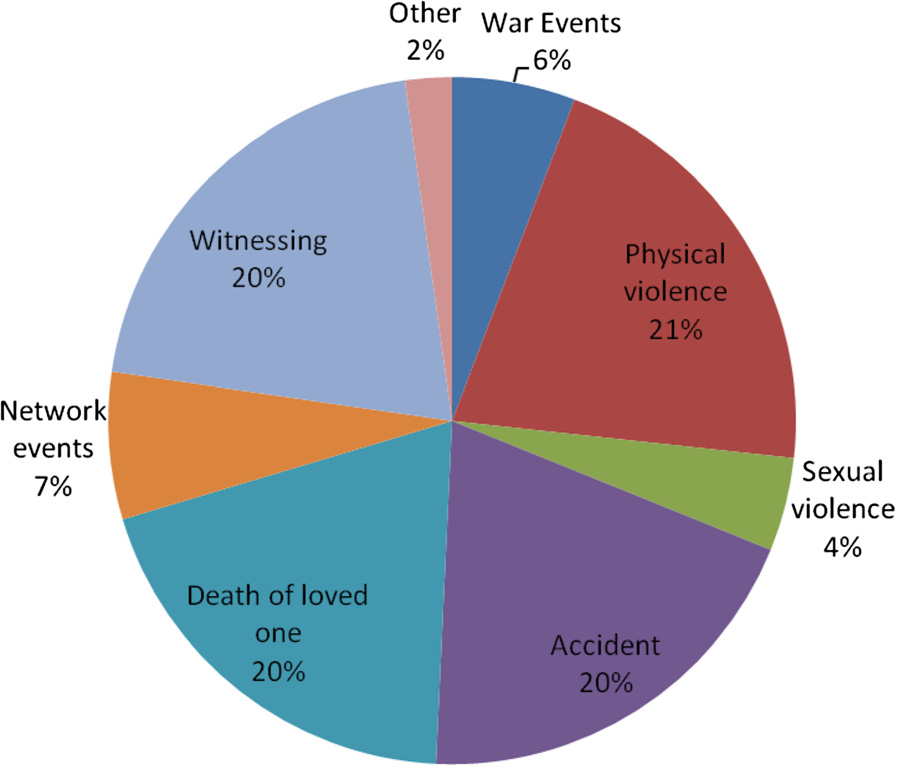
Traumatic events by category as percentage of all traumatics events.

Mean number of occurrences varied significantly across PTE classes (χ_7_ = 549.1, p < .001), resulting in the highest proportion of all lifetime PTEs being associated with physical violence (20.9%) followed by witnessing (20.5%), accidents (19.6%) and unexpected death of a loved one (19.6%) (Figure 1).

### Prevalence and conditional risk of PTSD

Conditional prevalence of PTSD refers to the prevalence of PTSD among those that had been exposed to PTEs, as opposed to the prevalence of PTSD in the total sample that includes those that were not exposed to any PTE.

The conditional prevalence of DSM-IV/CIDI PTSD after exposure to a PTE averages 3.5% (1.9) across all PTEs, for a total of approximately 480 lifetime episodes of PTSD (i.e., 3.5% of approximately 13,700 PTE occurrences) among SASH respondents. One or more lifetime episodes of PTSD was reported by 2.3% (0.3) of respondents, although this is an under-estimate to an unknown degree of the proportion of respondents with lifetime PTSD due to the fact that we did not assess PTSD for every PTE reported by every respondent. The prevalence estimate of 12-month PTSD, in comparison, is 0.7% (0.2).

As shown in Table 2, conditional risk for PTSD associated with PTE classes ranges from a high of 8.3% (4.9) associated with witnessing PTEs to a low of 1.4% (0.8) associated with network PTEs (i.e., PTEs that occurred to loved ones) but does not vary significantly across broad PTE classes (χ_7_ = 0.60, p = .75). Conditional PTSD risk varies even more across specific PTEs, with the highest value by far being associated with purposely injuring, torturing or killing someone (32.9%) and witnessing atrocities (31.5%). This variation is however not statistically significant (χ_27_ = 1.00, p = .44). The next highest conditional risks are associated with rape (13.2%) and life-threatening accidents (5.5% for auto accidents; 9.6% for other accidents). Conditional risks associated with the other 22 PTE types are considerably lower (0.0-5.4%).

**Table 2:** Conditional risk of DSM-IV/CIDI PTSD by PTE type, mean duration and relative PTSD burden associated with PTEs in the South Africa Stress and Health Study (n = 4315)

**Event type**	**Conditional PTSD risk**^**a**^	**SE**	**Number of lifetime to date PTSD episodes**^**b**^	**SE**	**Mean PTSD Duration (Months)**^**c**^	**SE**	**% relative PTSD burden**^**d**^	**SE**
**War events**	**2.9**	**2.80**	**0.5**	**0.52**	**70.7**	**10.77**	**8.1**	**7.53**
Combat	2.3	2.19	0.1	0.08	60.0	0.00	0.8	0.89
Relief worker	0.0	0.00	--	--	--	--	--	--
Civilian in war zone	0.0	0.00	--	--	--	--	--	--
Civilian in terror	0.0	0.00	--	--	--	--	--	--
Refugee	0.0	0.00	--	--	--	--	--	--
Purposely injured, tortured, or killed someone	32.9	28.73	0.5	0.18	72.0	0.00	4.3	1.68
**Physical violence**	**1.8**	**0.65**	**1.2**	**0.42**	**27.5**	**16.14**	**6.9**	**5.92**
Beaten up by caregiver	1.2	0.71	0.1	0.09	14.5	6.12	0.5	0.38
Beaten up by partner	5.4	2.69	0.4	0.22	59.0	44.20	5.3	5.34
Beaten up by someone else	0.9	0.82	0.2	0.15	2.0	0.00	0.10	0.12
Mugged or threatened with a weapon	1.7	0.98	0.4	0.26	11.1	6.06	1.1	0.84
Kidnapped								
**Sexual violence**	**2.6**	**1.70**	**0.4**	**0.24**	**15.2**	**12.39**	**1.2**	**1.21**
Raped	13.4	8.36	0.4	0.24	15.2	9.59	1.2	1.21
Sexually assaulted	0.0	0.00	--	--	--	--	--	--
Stalked	0.0	0.00	--	--	--	--	--	--
**Accidents**	**3.3**	**1.23**	**2.1**	**0.78**	**39.1**	**17.23**	**17.2**	**10.55**
Toxic chemical exposure	0.0	0.00	--	--	--	--	--	--
Automobile accident	5.5	3.57	0.9	0.60	33.7	14.88	6.3	4.39
Other life threatening accident	9.6	5.31	0.8	0.40	20.1	9.91	3.2	2.00
Natural disaster	0.7	0.73	0.0	0.02	7.0	0.00	0.1	0.03
Man-made disaster	0.5	0.65	0.0	0.06	72.0	0.00	0.3	0.89
Life-threatening illness	1.8	0.87	0.4	0.19	91.8	55.35	7.3	6.01
**Unexpected death of loved one**	**3.3**	**1.32**	**2.0**	**0.84**	**27.5**	**8.10**	**11.8**	**7.83**
**Network events**	**1.4**	**0.83**	**0.3**	**0.18**	**53.1**	**34.72**	**3.6**	**3.43**
Child with serious illness	1.9	1.36	0.2	0.15	68.8	21.45	3.0	3.26
Traumatic event to loved one	0.1	0.11	0.0	0.01	24.0	0.00	0.0	0.05
Accidentally caused serious injury or death	3.4	3.65	0.1	0.11	24.0	0.00	0.5	0.59
**Witnessing**	**8.3**	**4.91**	**4.5**	**2.46**	**55.1**	**4.38**	**50.5**	**15.38**
Witnessed death/dead body, saw someone hurt	3.7	2.67	2.1	1.54	44.1	9.51	19.8	13.92
Saw atrocities	31.5	18.34	2.4	1.37	61.6	1.42	30.7	10.82
**Others**	**2.5**	**1.91**	**0.2**	**0.14**	**21.4**	**8.19**	**0.8**	**0.61**
Some other event	0.9	0.91	0.0	0.01	48.0	0.00	0.3	0.15
Private event	1.2	0.13	4.1	3.77	1.4	1.28	0.5	0.48
**Total with any event**	**3.5**	**1.91**	**11.2**	**6.29**	**42.3**	**12.27**	**100.0**	**0.00**

^a^Conditional risk for PTSD varies significantly across broad PTE classes (χ_7_ = 134.2 p < .001) and even more across specific PTEs (χ_17_ = 356, p < .001). Some classes of events are not included in the comparison because their number of mean occurrences is equal to zero. ^b^Number of lifetime-to-date episodes of PTSD associated with this class of PTEs and individual PTE per 100 respondents. ^c^Mean duration of PTSD episode (or residual symptoms, in months) for episodes associated with PTE in this class (χ_7_ = 134.2, p < .001) and individual PTE types(χ_11_ = 416.1, p < .001). ^d^Percent of all years (or months) with PTSD episode (or residual symptoms) in the population due to episodes associated with this class of events.

Once PTSD occurs, PTSD duration varies significantly depending on the PTEs implicated in the PTSD (for PTE classes: χ_7_ = 134.2, p < .001; for individual PTEs: (χ_11_ = 416, p < .001). Mean duration (standard error) for all PTSD episodes is 42.3 (12.3) months. There is little precision in studying between-PTE variation in mean duration due to the small numbers of respondents with PTSD associated with specific PTEs. Focusing on PTE classes with 10 or more respondents, accidents and sudden unexpected death had a lower than average duration while the duration of PTSD related to witnessing events was higher than average.

The relative burden of PTSD refers to the percentage of all months (or years) lived with PTSD (or residual symptoms) in the population due to episodes associated with each class of events. The relative burden is a combination of three factors: the prevalence of the PTE, the conditional risk of PTSD following the PTE and the PTSD symptom duration.

For individual events, the highest relative burden is associated with unexpected death of a loved one (11.8%), witnessed death/dead body (19.8%) and saw atrocities (30.7%). Each of these events imposes high burden for different reasons.

For example, unexpected death of a loved one is one of the most common events with 39.2% prevalence. However, the probability of PTSD (conditional risk) associated with this PTE is only 3.3% while the duration of PTSD episodes associated with it is only 27 months. Therefore, despite the low conditional risk of PTSD and relatively short duration of symptoms compared to other events, unexpected death contributes to the total burden because it is very common. Seeing atrocities, on the other hand, is not a very common PTE, with only 3.9% of respondents reporting this event. However, the conditional risk of PTSD (31.5%) and duration (60.6 months) associated with this event are very high, resulting in this PTE’s high contribution to the total PTSD burden at almost one-third.

**Figure 2:**
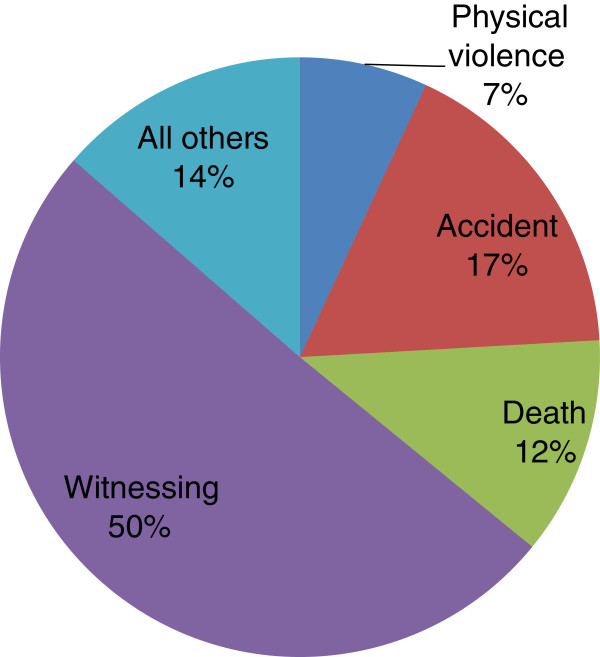
Relative PTSD burden associated with specific events in the South African population.

The PTE class which accounted for the largest relative burden of PTSD was witnessing: seeing atrocities, which was relatively rare at 3.9% but had a high conditional risk of PTSD (31.5%), and witnessing a death/dead body/someone hurt which was common (28.5%) but had a relatively low conditional risk of PTSD (3.7%). This PTE class had a longer than average duration of symptoms with the overall duration for witnessing being 55.1 months. Figure 2 illustrates that witnessing events account for over 50% of the burden of PTSD in this population.

### Socio-demographic predictors of trauma exposure, lifetime and 12-month PTSD

Trauma exposure, lifetime and 12-month PTSD were predicted by few socio-demographic factors (Tables 3 and 4 respectively). Compared to employed participants, homemakers were at significantly lower risk of exposure to PTEs (OR = 0.65, CI 0.49-0.86).

**Table 3:** Associations of socio-demographic factors with lifetime PTSD in the South Africa Stress and Health Study (N = 4315)

**Variable**	**Lifetime trauma exposure OR (OR range)**	**Lifetime PTSD OR (OR range)**		
		**Among total sample**	**Among those with events**	**Among those with events, Controlling for events**
**Gender**				
Male	1.00	1.00	1.00	1.00
Female	1.03 (0.84-1.26)	1.24 (0.56-2.76)	0.53 (0.12-2.30)	0.84 (0.34-2.04)
**Age**				
18 - 29	0.89 (0.64-1.25)	1.24(0.39-3.90)	0.76 (0.14-4.27)	1.70 (0.35-8.26)
30 - 44	1.04 (0.72-1.5)	1.62 (0.60-4.39)	0.59 (0.11-3.08)	0.83 (0.15-4.69)
45 - 59	1.22 (0.89-1.67)	1.69 (0.69-4.15)	0.51 (0.12-2.09	0.91 (0.25-3.36)
60+	1.00	1.00	1.00	1.00
**Marital status**				
Married	1.00	1.00	1.00	1.00
Previously married	1.35 (0.85-2.14)	2.71 (1.16-6.35)*	0.36 (0.09-1.36)	0.47 (0.10-2.28)
Never married	0.85 (0.67-1.08)	0.74 (0.33-1.66)	0.80 (0.30-2.14)	0.66 (0.20-2.16)
**Education level**				
Low	0.59 (0.38-0.94)	0.47 (0.18-1.23)	0.71 (0.10-4.92)	0.68 (0.10-4.92)
Low-average	0.72 (0.48-1.06)	0.70 (0.38-1.26)	3.16 (0.66-15.19)	1.92 (0.65-5.66)
High-average	0.85 (0.65-1.12)	0.55 (0.29-1.06)	0.76 (0.22-2.64)	0.31 (0.08-1.23)
High	1.00	1.00	1.00	1.00
**Employment**				
Employed	1.00	1.00	1.00	1.00
Homemaker	0.65 (0.49-0.86)*	1.00 (0.40-2.52)	1.15 (0.29-4.58)	0.92 (0.26-3.24)
Retired	0.87 (0.54-1.39)	2.33 (0.89-6.09)	11.97 (4.00-35.82)*	9.18 (2.62-32.20)*
Other	0.86 (0.67-1.10)	1.14 (0.70-1.87)	1.23 (0.56-2.72)	1.06 (0.50-2.24)
**Race**				
White	1.00	1.00	1.00	1.00
Black	1.32 (0.81-2.16)	0.70 (0.29-1.68)	0.36 (0.10-1.28)	0.60 (0.23-1.61)
Colored	0.73 (0.42-1.25)	0.90 (0.50-1.62)	0.76 (0.14-4.01)	0.79 (0.21-3.06)
Indian	1.35 (0.69-2.62)	0.07 (0.01-0.57)*	0.01 (0.00-0.09)*	0.02 (0.00-0.19)*
Other	1.17 (0.51-2.71)	0.95 (0.22-4.09)	0.61 (0.04-9.16)	1.23 (0.18-8.41)
**PTE classes**				
War events	-	-	-	2.40 (0.32-18.06)
Physical violence	-	-	-	0.68 (0.23-2.00)
Sexual violence	-	-	-	0.74 (0.08-7.32)
Accident	-	-	-	1.43 (0.31-6.55)
Death	-	-	-	1.00
Network events	-	-	-	0.38 (0.06-2.32)
Witnessing	-	-	-	3.56 (0.53-24.03)
Other or private	-	-	-	0.74 (0.09-6.40)
**Count of prior PTEs**				
Prior war events	-	-	-	0.58 (0.29-1.15)
Prior physical violence	-	-	-	1.33 (0.73-2.43)
Prior sexual violence	-	-	-	1.56 (0.91-2.69)
Prior accidents	-	-	-	0.95 (0.73-1.23)
Prior deaths	-	-	-	1.53 (1.16-2.03)
Prior network events	-	-	-	1.10 (0.77-1.57)
Prior witnessing	-	-	-	1.09 (0.93-1.28)
Prior other events	-	-	-	0.14 (0.01-2.92)

*Statistically significant (p < 0.05); *OR* Odds Ratio.

**Table 4:** Associations of socio-demographic factors with 12-month PTSD in the South Africa Stress and Health Study (N = 4315)

**Variable**	**12-month PTSD in total sample; OR (OR range)**	**12-month PTSD among those with events; OR (OR range)**	**12-month PTSD among those with events, controlling for events; OR (OR range)**	**12-month PTSD among those with lifetime PTSD; OR (OR range)**
**Gender**				
Male	1.00	1.00	1.00	1.00
Female	1.55 (0.58-4.14)	0.44 (0.06-3.27)	0.45 (0.12-1.68	2.40 (0.32-17.82)
**Age**				
18 - 29	0.95 (0.10-9.50)	4.60 (0.17-123.08)	2.86 (0.16-50.34)	11.29 (0.17-743.84)
30 - 44	1.43 (0.35-5.86)	3.66 (0.61-21.89)	2.58 (0.43-15.50)	10.57 (0.29-384.73)
45 - 59	1.21 (0.23-6.42)	4.82 (0.47-49.80)	4.81 (0.61-37.88)	21.85 (0.66-722.73)
60+	1.00	1.00	1.00	1.00
**Marital status**				
Married	1.00	1.00	1.00	1.00
Previously married	1.34 (0.38-4.71)	0.19 (0.01-2.72)	0.30 (0.06-1.62)	0.02 (0.00-0.31)*
Never married	0.78 (0.20-3.02)	1.70 (0.49-5.92)	3.76 (0.78-18.15)	37.64 (2.22-639.50)*
**Education level**				
Low	0.14 (0.01-1.42)	0.49 (0.03-9.92)	0.59 (0.03-13.83)	.
Low-average	1.56 (0.38-6.38)	4.56 (0.30-68.37)	5.55 (0.29-106.30)	29.45 (2.62-330.50)*
High-average	0.53 (0.14-2.01)	0.17 (0.03-0.91)*	0.10 (0.01-0.82)*	2.03 (0.18-23.31)
High	1.00	1.00	1.00	1.00
**Employment**				
Employed	1.00	1.00	1.00	1.00
Homemaker	0.67 (0.25-1.75)	0.24 (0.05-1.29)	0.41 (0.09-1.94)	.
Retired	0.44 (0.06-3.20)	0.14 (0.02-1.14)	0.14 (0.02-1.15)	.
Other	1.17 (0.47-2.92)	0.49 (0.14-1.75)	0.68 (0.22-2.07)	0.15
**Race**				
White	1.00	1.00	1.00	.
Black	1.05 (0.20-5.54)	13.38 (0.90-199.26)	14.97 (0.71-318.08)	.
Colored	1.08 (0.23-5.06)	11.38 (0.75-172.24)	16.86 (0.55-518.03)	.
Indian	0.40 (0.03-4.95)	0.77 (0.03-19.87)	2.37 (0.04-155.10)	.
Other	4.45 (0.33-60.76)	42.19 (0.83-2138.38)	27.14 (0.72-1024.36)	.
**PTE classes**				
War events, physical or sexual violence	-	-	0.95 (0.20-4.51)	0.33 (0.05-2.29)
Accident	-	-	1.21 (0.19-7.68)	0.73 (0.07-7.91)
Death	-	-	1.00	1.00
Network events	-	-	0.03 (0.00-1.95)	0.13 (0.01-2.04)
Witnessing	-	-	2.04 (0.38-10.75)	0.09 (0.00-2.12)
Other or private	-	-	0.10 (0.00-2.38)	3.39 (0.03-430.50)
**Count of prior PTEs**				
Prior war events	-	-	0.25 (0.03-1.89)	1.12 (0.25-5.06)
Prior physical violence	-	-	1.53 (0.96-2.45)	2.43 (0.98-6.06)
Prior sexual violence	-	-	2.38 (1.21-4.69)	2.97 (0.98-8.99)
Prior accidents	-	-	1.32 (0.99-1.76)	0.87 (0.33-2.33)
Prior deaths	-	-	0.57 (0.23-1.42)	1.08 (0.55-2.11)
Prior network events	-	-	0.12 (0.01-2.08)	0.05 (0.01-0.28)
Prior witnessing	-	-	1.09 (0.88-1.35)	3.38 (1.38-8.27)
Prior other events	-	-	0.40 (0.03-5.69)	89.72 (0.91-8832.05)

*Statistically significant (p < 0.05); *OR* Odds Ratio.

Low and low-average education was combined in the model predicting 12-month PTSD among respondents with LT PTSD; Homemaker, retired and other employment categories were combined in the model predicting 12-month PTSD among respondents with LT PTSD.

For lifetime PTSD, Indians had reduced odds (OR = 0.02, CI 0.00-0.09) and retired persons increased odds (OR = 9.18, CI 2.62-32.20) in fully adjusted models. For 12-month PTSD among those with lifetime PTSD, previously married participants had lower odds (OR = 0.02, CI 0.00-0.31) compared to currently married respondents, whereas the never-married had significantly elevated odds (OR = 37.64, CI 2.22-639.50). Compared to those with high education, those with low-average education had significantly increased odds of having 12-month PTSD (OR = 29.45, CI 2.62-330.50).

## Discussion

The present study goes beyond previous South African work in several significant ways. First, we present the first national estimates of pervasive trauma exposure across the full range of PTEs. Over 70% of the population was exposed to at least one PTE, a prevalence rate similar to that found in two urban US epidemiologic samples using the random event method where rates of 89.6% and 87.2% respectively were reported [10,19]. The most striking finding was that unexpected death of a loved one and witnessing death or seeing a dead body or someone getting seriously hurt accounted for over two fifths of all reported PTEs. This is new information since prior studies in South Africa have suggested the predominance of interpersonal violence over other types of trauma exposure [20,21]. However, these studies did not assess the full range of exposures, possibly leading to an under-estimation of the importance of unexpected death and witnessing.

Second, a surprising finding was a lifetime PTSD prevalence of 2.3% in South Africa, significantly lower than rates found in European (7.4%) and North American (6.8%) studies [22,23]. The PTSD conditional risk of 3.5% is also significantly lower than that found in previous work using the random event method, where Breslau et al described a PTSD conditional risk of 9.2% [10]. In particular, the conditional risk of PTSD in relation to sexual assault was considerably lower than the 30-50% observed in most other epidemiologic studies [10,12,24]. Potential explanations for cross-national differences in PTSD prevalence are numerous and deserve further study. One possibility to note is the impact of the PTSD diagnostic criteria requiring avoidance of traumatic reminders. The public nature of political and criminal violence in South Africa may have made avoidance difficult, thereby attenuating our estimates of PTSD prevalence. It has been suggested elsewhere that the avoidance criterion for PTSD may be too stringent in certain cases, resulting in sub-threshold PTSD that causes as much functional impairment as full PTSD [25]. Future studies could examine this possibility by estimating the prevalence of specific PTSD symptom clusters and sub-threshold PTSD in South Africa. Similar studies from other countries report significant rates of sub-threshold PTSD that are, in some cases, even higher than full PTSD [26,27].

Third, witnessing events – many of which likely included violence – accounted for 50% of the relative PTSD burden and were associated with a very long duration of symptoms, second in chronicity only to symptoms associated with war events. These findings contrast sharply with results from other populations where the unexpected death of a loved one [10] and direct interpersonal violence (rape and combat) [12] are associated with the largest proportion of PTSD episodes. This may reflect the fact that political and criminal violence often occurs in public settings in South Africa, and highlights the importance of the political and social context in shaping the risk of PTSD related to specific events [9]. The prominent role witnessing PTEs plays in PTSD causation in South Africa may be related to the culturally prescribed linkage of one’s well-being to the well-being of one’s family and community. This philosophy of *ubuntu* has been described as an African world-view that emphasises “group solidarity, conformity, compassion, respect, human dignity, humanistic orientation and collective unity” [28]. Alternatively, compared to directly experiencing a traumatic event, witnessing may have differential effects on memory and feelings of helplessness that may be important in PTSD aetiology. Indeed, it has been argued that “the impact of witnessing trauma is likely to be more distressing for individuals who have experienced multiple traumas. The witnessing experience may have more impact on individuals who are sensitized to trauma through enhancing memory formation; thus, intrusive and vivid recall is more likely” [29]. Our findings are also consistent with prior empirical work on the aversive effects of watching the infliction of pain on others, and with prior studies of PTSD in high-risk groups such as war journalists and rescue workers [30,31]. Studies focused on individuals’ direct experiences of violence are therefore likely to underestimate the burden of PTSD in countries like South Africa that have experienced extreme political and social conflict with frequent public perpetration of violence.

Finally, we found little association between socio-demographic predictors and PTEs, lifetime or 12-month PTSD in the South African population. For instance, many epidemiologic studies in other civilian populations have consistently documented that men are at increased risk of trauma exposure while women experience increased risk of lifetime PTSD [12,32,33]. Neither of these findings was observed in our study. It is possible that this lack of association between PTSD and most socio-demographic factors is due to the unique history of trauma exposure in South Africa, where institutionalised violence and traumatisation were common features during the *apartheid* regime, with almost the entire population being exposed at some point [1]. Consistent with our findings, previous work among South African school-children failed to demonstrate any association between PTSD symptoms and gender, although there were mixed associations with different types of trauma [5]. This study by Seedat et al [5] further demonstrates that even post-apartheid, exposure to severe PTEs is still common in South Africa, even if the nature of traumatisation has shifted from a political to a largely criminal nature. Similarly, a recent study of survivors of Hurricane Katrina in the US demonstrated that the hurricane-related trauma exposure was so pervasive that typical socio-economic and other demographic associations with PTSD were not observed [34]. However, some studies from elsewhere in Africa, such as in South Sudan, Rwanda and Uganda continue to show associations between PTSD and sociodemographic variables such as gender and marital status [35-37]. Further research is necessary to explain the nature of these differences, some of which may be attributed to methodological variations. In our study, the few associations we found between sociodemographic characteristics such as education and marital status (with 12-month PTSD) and employment status (with Lifetime PTSD) were characterised by very wide confidence intervals due to the very limited number of respondents in each category. A study specifically targeting these associations may provide useful information that may inform future interventions in these and other settings.

The major limitation of this study is that, as in all general population studies of trauma and PTSD, we rely on participants’ retrospective reporting of PTEs and symptoms. Firstly, respondents may be more likely to report PTEs that caused significant distress, potentially under-reporting other PTEs, although our use of a detailed trauma checklist should reduce that problem. Secondly, it is possible that participants underreported stigmatized PTEs such as sexual assault. Previous work by Jewkes and others [3,38-40] highlights the complexity of power relations and sexual transactions in South Africa that affect the experience and reporting of sexual abuse. Thirdly, we depended on respondents’ respective recall of the timing of PTEs and subsequent emergence of PTSD symptoms. Retrospective recall of mental disorders, particularly over long intervals, tends to underestimate the true prevalence of these disorders [41,42]. Our prevalence estimates of PTSD are therefore likely to be conservative.

## Conclusions

In this study, we have demonstrated the ubiquitous nature of sudden unexpected deaths and witnessing of traumatic events in South Africa, and the contribution of these events to the national PTSD burden. A cross-cutting theme from these findings is that interventions aimed at reducing PTSD in the South African population will need to increase the focus on special groups that have not traditionally received much attention after traumatic events- observers, retirees and bystanders during the occurrence of traumatic events. The traditional focus on direct victims has left out these important groups, and it is clear that reducing the occurrence of PTSD among these groups will significantly reduce the overall burden of PTSD in the general population.

## Competing interests

LA, KAM, MP, and KCK have no competing interests.

DJS has received research grants and/or consultancy honoraria from Abbott, AstraZeneca, Eli-Lilly, GlaxoSmithKline, Jazz Pharmaceuticals, Johnson & Johnson, Lundbeck, Orion, Pfizer, Pharmacia, Roche, Servier, Solvay, Sumitomo, Takeda, Tikvah, and Wyeth.

RCK has been a consultant for GlaxoSmithKline, Kaiser Permanente, Pfizer, Sanofi-Aventis, Shire Pharmaceuticals and Wyeth-Ayerst; has served on advisory boards for Eli Lilly & Company and Wyeth-Ayerst; and has had research support for his epidemiological studies from Bristol-Myers Squibb, Eli Lilly & Company, GlaxoSmithKline, Johnson & Johnson Pharmaceuticals, Ortho-McNeil Pharmaceuticals, Pfizer and Sanofi-Aventis.

## Authors’ contributions

DJS, DRW, KAM, MP, RCK and KCK were involved in all stages of the design and implementation of the study. All authors participated in data analysis and drafting of the manuscript, led by LA. All authors read and approved the final manuscript.

## Pre-publication history

The pre-publication history for this paper can be accessed here:

http://www.biomedcentral.com/1471-244X/13/182/prepub
